# One Health genomic surveillance of ticks found on community-dwelling non-human ‘farm’ animals to identify microbes associated with tick borne illnesses in Eastern Ontario

**DOI:** 10.1371/journal.pone.0359215

**Published:** 2026-10-09

**Authors:** L. F. Carver, S. Chesney, S. Kannurpatti Srinivasan, D. Bourne, R. Colautti

**Affiliations:** 1 Faculty of Health Science, Queen’s University, Kingston, Ontario, Canada; 2 Biology Department, Queen’s University, Kingston, Ontario, Canada; National Veterinary Research Institute (NVRI), NIGERIA

## Abstract

Companion animals, such as dogs and cats, and wildlife are recognized as sentinel species in One Health surveillance due to periodic exposure to ticks and infection by tick-borne pathogens (TBPs). Surveillance of this type is a strategy that captures a broad range of genetic, immunological, and behavioral factors that affect tick and disease dynamics. In contrast, non-human animals (hereafter referred to as ‘animals’) that are traditionally farmed for their meat and other products have been largely overlooked as sentinel species. Community-dwelling farm sanctuary residents, such as cattle, pigs, horses, and other traditionally ‘farmed’ animals have close daily contact with human caregivers, making them excellent candidates for One Health surveillance. These animals, often free-range in pastures and/or woodlands, bridging the territory inhabited by wildlife and that occupied by humans. This study collected 67 ticks, from bovines (n = 22), equines (n = 35) and canines (n = 6) residing in a farm animal sanctuary and screened them for microbes associated with tick borne illnesses using 16S rRNA V4 amplicon targeted sequencing (515F/906R) with custom *Rickettsia buchneri* blocking primers to boost sensitivity. Only 60 ticks were viable for analysis, 59 were of *Ixodes* species and one was *Dermacentor variabilis.* Of these, 31.6% (n = 19) tested positive for potential pathogens, including 21.6% (n = 13) for the causative agent of Lyme disease (*Borrelia burgdorferi*) and 15% (n = 9) for *Anaplasma phagocytophilum*, with a co-infection rate of five percent (n = 3). Utilizing community dwelling sanctuary residents allowed us to detect potential threats to both animal and human health and was instrumental in the rapid diagnosis of anaplasmosis in two study participants (a horse and a pony) who showed symptoms of lethargy and inappetence after being bitten by ticks.

## Introduction

Zoonoses are diseases transmitted between humans and wild or domestic animals, accounting for sixty to seventy-five percent of all known and emerging infectious diseases [1,2]. Vector-borne diseases, in particular, pose a significant health threat, accounting for approximately 17% of infectious diseases and being implicated in more than 700,000 human deaths annually [3,4]. Of these, ticks transmit approximately 95% of vector-borne diseases in the United States, however, only 25 of the hundreds of species of ticks identified around the world are considered significant threats to humans and animals [5,6]. Although mosquitoes are more prevalent, ticks transmit a more diverse array of bacterial, protozoan, and viral pathogens than other blood-feeding arthropods [5,7]. Ixodid or hard-bodied ticks, detected in both natural and urban environments, are a particular concern, with prolonged host contact and extended feeding periods typically ranging from 3–12 days, increasing the efficiency of pathogen transmission and, thus, the burden of tick-borne disease [6,8].

The One Health approach to zoonoses recognizes that health risks involve complex interactions among host species, vectors, infectious agents, and the environment [1,3]. Guided sentinel surveillance involves repeated sampling from within populations, including animals, in a multisectoral collaboration, facilitating an interdisciplinary active surveillance method contrasting with the commonly siloed approach to health [9,10]. Passive surveillance is considered a more resource-efficient method than active surveillance. Active tick surveillance is generally done by collecting samples from hosts or using methods such as drag sampling [9]. When active surveillance occurs, as was done in this study, it allows for the identification of sentinel species that develop clinically or immunologically measurable responses to infectious disease. These sentinel species are valuable as they may help identify “hot spots” potentially enabling earlier pathogen detection and insights into changes in prevalence, incidence, or geographic distribution [10].

Wild animals, having co-evolved with ticks and tick-borne pathogens, act as reservoirs or amplifying hosts for infectious agents, supporting the maintenance of endemic disease cycles [11–13]. These species migrate within and between green spaces in urban or suburban areas, without being fully restricted by natural or man-made barriers [12,13]. The close proximity increases the frequency of interactions between tick-carrying or infected wildlife and humans or domestic animals [12]. Examining seroprevalence or identifying infected ticks carried by wild animals highlights pathogen presence, identifies areas of risk of exposure, and supports mapping locations for future collection and study [12,14].

Companion animals such as dogs, may also be effective sentinels due to their close proximity to humans and cohabitation [15]. Some estimates suggest that dogs in endemic areas encounter infected ticks at approximately six times the rate of their human caregivers [15]. In areas where tick species such as *Ixodes scapularis* are established, serologic sampling of these dogs may provide valuable information about the risk of tick-borne disease [10,15,16]. Dogs may transport ticks into closer proximity to their caregivers after exposure, thereby establishing tick populations near or within human dwellings [10,17]. Infestations and pathogen transmission may occur following the introduction of infected larvae or nymphs after molting or following the introduction of engorged females, which lay eggs following post-blood meal detachment from hosts [17,18].

Tick population densities are generally highest in regions with high host availability and favorable habitats or microclimates. Pastures, which often contain wooded areas, may be important for investigating the presence of ticks or tick-borne pathogens affecting humans, wildlife, and domestic animals [19–22]. In rural settings where domestic animals live in pastures or mixed farmland which overlaps with the natural habitats of wildlife, the diversity of species promotes the circulation of tick-borne pathogens in the environment. Cattle, equines, and other commonly ‘farmed’ animals, as well as companions such as cats or dogs, and wild animals serve inroles ranging from dilution and amplifying hosts to competent reservoirs [20]. They may, in fact, serve essential roles in these environments. Transport of ticks from woodlands to areas of human and domestic animal use may be essential for maintaining tick populations, especially since approximately 48–100% of farms in highly affected regions report the presence of ticks [20,21]. Notably, direct and indirect contact between domestic animals and pathogen-carrying wildlife is facilitated in free-range systems thereby contributing to the maintenance or transmission of tick-borne and previously isolated infectious agents and increasing the risk of human exposure [20,23]. Because free-ranging domestic animals may provide earlier insights into tick presence or associated impacts, they are valuable in sentinel surveillance efforts [15,20,23].

Current approaches to tick-borne pathogen surveillance rely primarily on targeted assays, such as qPCR and serology, designed to detect specific pathogens of interest. While sensitive and specific, targeted methods cannot detect novel, emerging, or unexpected pathogens, miss rare variants due to primer mismatches, and require multiple separate assays to screen for co-infections [24]. A key motivation for moving beyond single-pathogen-targeted assays is the risk of co-infection – the simultaneous presence of multiple tick-borne pathogens within a single tick or host. *I. scapularis* ticks can carry several pathogens simultaneously, and because these pathogens share reservoir hosts, co-infection rates in ticks are higher than would be expected by chance alone [25–27]. In humans, co-infections with tick-borne pathogens are associated with more severe symptoms, longer illness duration, and a greater likelihood of treatment failure than single-pathogen infections [26,28,29]. However, standard diagnostic protocols typically focus on a single pathogen of concern – most commonly *B. burgdorferi*, potentially missing concurrent infections and complicating clinical management. Understanding the prevalence of co-infection among ticks removed from animals in close contact with humans is both a surveillance and a clinical priority.

High-throughput sequencing (HTS) of the 16S rRNA gene offers a complementary approach that detects multiple co-infections or bacterial taxa, including pathogens, endosymbionts, and novel microbes, in a single assay [30–34]. Applying this approach to *I. scapularis* ticks in Southeastern Ontario, Canada, revealed co-infections by *Borrelia spp*., *Borrelia miyamotoi*, and *Anaplasma phagocytophilum* that standard targeted protocols may miss, offering a surveillance tool better suited to the multi-pathogen reality of tick-borne disease risk in other regions of Ontario [30]. Advances in sequencing platforms have further expanded HTS, with nanopore-based metagenomics now enabling targeted enrichment of tick-borne pathogen sequences from field-collected ticks [35,36], and broader metagenomics-based surveillance becoming increasingly feasible as costs decline [37]. Despite these advances, HTS is not routinely used for tick surveillance in Canada, and its potential for One Health applications — including screening ticks collected from community-dwelling animals — remains largely unrealized [30].

A key technical barrier to HTS-based surveillance in *I. scapularis* is the dominance of *Rickettsia buchneri*, a maternally transmitted endosymbiont found across all *I. scapularis* tissues but at particularly high abundances in the reproductive tissues of adult females [30,38,39]. *R. buchneri* is believed to supplement the tick’s blood-meal diet by synthesizing biotin and other nutrients deficient in vertebrate blood, and its relative abundance commonly exceeds 90% of 16S rRNA reads in adult female *I. scapularis* samples [34,40–42]. This dominance effectively crowds out the signal from low-abundance pathogens such as *B. burgdorferi* and *A. phagocytophilum*, reducing the sensitivity of 16S amplicon sequencing for pathogen detection. Researchers previously established a proof of concept for addressing this problem in *I. holocyclus* ticks from Australia by using an annealing-inhibiting blocking primer targeting the endosymbiont *Candidatus Midichloria mitochondrii*, which reduced endosymbiont reads by ~96% and enabled detection of multiple pathogens previously masked by the dominant taxon [43]. We extended this approach to *I. scapularis* by developing and validating a blocking primer targeting *R. buchneri* [44]. In the present study, this blocking primer was used as part of a three-step amplicon library preparation protocol to screen ticks collected from community-dwelling animals at a farm animal sanctuary in Eastern Ontario.

### Rationale for this study

This study was designed to test the efficacy of the screening technique described above and answer three main questions. First we wanted to know what type of ticks are found on domestic animal hosts in this region of Eastern Ontario. Second, we wanted to find out which pathogens are found in the ticks collected in this region. Specifically, are these limited to the two vector-borne pathogens tested by Public Health Ontario (*Borrelia burgdorferi* and *Anaplasma phagocytophilum*), or are there other pathogens in these Eastern Ontario ticks that would not be detected by current public health surveillance? And, finally, we were interested in whether single ticks commonly carry more than one pathogen?

## Methodology

### Sample collection and DNA extraction

From May 19^th^ to September 27^th^, 2023, 67 ticks were collected from community-dwelling non-human animals (CD-NHA) residing at a farm animal sanctuary in Eastern Ontario. Farm sanctuaries are a type of animal rescue that can include animals such as cattle, pigs, horses, poultry as well as the usual companion animals, dogs and cats, among their NHA residents. The CD-NHAs are often in close contact with human caregivers who tend to engage in patting, scratching, and hugging, as well as caregiving activities such as providing food, water, and bedding changes. As a result, the risk of a tick crawling from sanctuary residents to a human caregiver in these settings is high, much as it is with companion animals such as dogs and cats. Daily health checks were performed on all sanctuary residents including cattle, horses, ponies, dogs, cats and pigs. No ticks were found on the pig residents of the sanctuary during the collection period. However, 22 ticks were found on the bovine, 35 on the equine and six on the canine residents. For this study any ticks found during the daily health checks, attached or crawling on the CD-NHAs, were removed and placed in 2 mL screw-cap tubes containing 70% ethanol, which were then labeled using baRcodeR [45] with the sample date and the species on which the tick was found. The ticks were morphologically identified, transferred to 1.7 mL Eppendorf tubes containing 70% ethanol, labeled with a unique barcode identifier using baRcodeR [45], and stored at −80 °C until DNA extraction.

Prior to DNA extraction, ticks were surface sterilized with 1% sodium hypochlorite for 30 seconds and washed twice with nuclease-free water to remove surface microbial contaminants. We followed the DNA extraction protocol using cetyltrimethylammonium bromide (CTAB). After surface sterilization, the ticks were air-dried for 2 min and transferred to 2 mL sterile screw-cap tubes containing ZrO beads (low-binding SPEX) of sizes 0.2 mm and 0.5 mm in equal volumes. liquid nitrogen for 3 minutes. After freezing in liquid nitrogen for 3 minutes the samples were pulverized using the Next Advance Bullet Blender Storm 24 device at 100 Hz for 5 minutes. Next, we added 500 μL of preheated cetyltrimethylammonium bromide (CTAB) buffer (100 mM Tris-HCl [pH 8.0], 25 mM EDTA, 1.5 M NaCl, 3% CTAB, 1% polyvinylpyrrolidone, and 1% β-mercaptoethanol) to all the pulverized samples and incubated the tubes at 62 °C. After overnight incubation, we added 500 µL of chloroform and centrifuged for 15 min at 1500 × *g* to isolate genomic DNA. The supernatant was transferred to a 1.7 mL tube, and genomic DNA was precipitated by adding 2 volumes of ice-cold 100% ethanol, followed by mixing by inversion and incubation at −20 °C for 30 minutes. The samples were centrifuged at 21,000 × *g* for 15 minutes, washed twice with 1 mL of 75% ethanol, and resuspended in 15 µL of nuclease-free water. Total genomic DNA was quantified in the DS-11 FX spectrophotometer (DeNovix) and 1.5% gel electrophoresis.

### Library preparation and illumina sequencing

Amplicon library preparation and sequencing were performed as described previously [30], with modifications to improve sensitivity. Prior to library preparation, the genomic DNA samples were size-selected using homemade SPRI beads (1.2 × volume) to remove fragments shorter than 100 bp. Library preparation was performed in a three-step PCR, where PCR1 was used to amplify the full-length 16S rRNA sequences using primers 27F-YMY and 1492R-HY [46,47], along with a blocking primer in 7-fold molar excess (18F-Rb-C3) to suppress the amplification of intracellular endosymbiont *R. buchneri*.

As discussed in detail elsewhere, “blocking primer sequences were designed 132 from alignments of partial 16S sequences from common bacterial TBPs (e.g., *B. burgdorferi* 133 *sensu stricto*, *Anaplasma phagocytophilum*) and *R. buchneri*, retrieved from GenBank” [44]. The total reaction volume of PCR1 was 50 µL, containing 25 µL of Q5 High-Fidelity DNA Master mix (New England BioLabs), 0.4 µM of 27F-YMY and 1492-HY primers, 2.8 µM of 18F-Rb-C3 blocking primer, 3 μL total genomic DNA (~ 5–6 ng µL^-1^), and nuclease-free water to volume. PCR1 products were purified using homemade SPRI beads at 1 × volume to remove primer dimers and were eluted in 10 µL nuclease-free water. To amplify the V4 regions, PCR2 was performed using 515F-806R primers [48,49] and spacers, with a total reaction volume of 8 µL comprising 3 µL of PCR1-purified product, 4 µL of Q5 High-Fidelity DNA Mastermix (New England BioLabs), and 0.4 µM of each primer. The PCR2 product was diluted 1:10 with nuclease-free water and used as a template in PCR3. PCR3 was performed to add the Illumina i5 and i7 index primers to the amplicons. The total reaction volume is 10 µL, which includes 3 µL of diluted PCR2 product, 1 µM each of i5 and i7 primers, and 5 µL of Q5 High-Fidelity DNA Mastermix (New England BioLabs). All PCR amplification conditions are described previously [30]. Indexed PCR3 products were normalized using the Just-a-Plate™ 96 PCR purification and normalization Kit (JN-120–10; Charm Biotech) according to the manufacturer’s protocol and pooled libraries were eluted in 20 µL of nuclease-free water. In addition, the pooled libraries were size-selected using BluePippin (Sage Science) and eluted in 10 µL elution buffer. Final pooled libraries were quantified using the NEBNext Illumina library quantification kit according to the manufacturer’s protocol. Paired-end sequencing was performed at the Infectious Disease Sequencing Lab in the Kingston Health Sciences Center using the Illumina MiSeq platform with a MiSeq reagent kit v2 (500 cycles), producing 2 × 250 paired-end sequencing data.

## Bioinformatic and statistical analysis

i) Preprocessing, generating Amplicon Sequence Variants (ASVs) and removal of contaminants:

The following bioinformatic workflow was used: Raw paired-end libraries generated from the Illumina platform were converted and demultiplexed into individual FASTQ files using bcl2fastq v2.2.0 with default settings. The libraries were then preprocessed with cutadapt v 5.1 [50] to trim and remove primer sequences and low-quality bases (i.e., quality scores below 20). The preprocessed libraries were then analyzed using a variety of R v.4.4.1 packages in RStudio v.2025.09.0 [51,52]. Core sample-inference denoising algorithms were deployed on the libraries using DADA2 v.1.30.0 [53]. Following denoising, the forward and reverse libraries were trimmed to lengths of 210 and 200, respectively, merged, and the sequences were clustered into representative ASVs and screened for chimera using the consensus method. Taxonomic assignment of the representative ASVs was performed using the Greengenes2 v.2024_09 naïve Bayes classifier with the DADA2-formatted species-level training set (downloaded in 2026) [54,55]. The count and taxonomy tables generated by DADA2 were imported into a *phyloseq* object using *phyloseq* v.1.50.0 [56], and downstream analyses were performed.

Using *decontam* v.1.26.0 [57] and no-template controls from DNA extraction and library amplification, we removed contaminants using the prevalence method with a threshold value of 0.40 (*p = 0.40*). Furthermore, we removed the ASVs assigned to the taxa Chloroplast and Mitochondria. Finally, prevalence and abundance filtering were performed to remove spurious taxa. Taxa with a read count of fewer than 100 in a sample were removed, and taxa present in fewer than 2 samples were discarded. Negative controls and PCR controls were excluded based on sample name, and samples with fewer than 1,000 total reads were pruned. The resulting *phyloseq* object was used for microbial ecology analyses.

ii) Community ecology analyses:

To visualize the taxonomic ranks, we used *microViz* v.0.13.0 [58]. Prior to diversity analyses, a rarefaction curve was generated to identify the point at which microbial richness is saturated. After finding the optimal values, as previously suggested [59], we used repeated subsampling without replacement at a depth of 12000 for alpha analyses using the RStudio *vegan* package [60]. Alpha diversity was quantified using Hill numbers, which provide a unified framework for describing diversity: q = 0 corresponds to observed species richness, q = 1 to the exponential Shannon index (exp (Shannon)), and q = 2 to the inverse Simpson index. These metrics collectively capture richness (q = 0) and evenness-weighted diversity (q = 1, q = 2), and the mean values were used to visualize the box plot with *ggplot2* v.4.0.2 [61]. Distributions were assessed for normality using the Shapiro-Wilk test; because they were significant, nonparametric tests were applied. Pairwise comparisons between two groups were performed using the Wilcoxon rank-sum test, and comparisons among three or more groups were performed using the Kruskal-Wallis test followed by post hoc pairwise Wilcoxon tests with a Bonferroni correction. Effect sizes were estimated using the rank-biserial correlation coefficient. All alpha diversity statistical analyses were performed using *rstatix* v.0.7.3 [62], and results were visualized as boxplots using *ggplot2* v 4.0.2 and *ggpubr* v.0.6.3 [61,63]. Similarly, for beta diversity, Bray-Curtis and UniFrac distance matrices were constructed using repeated subsampling without replacement, with a depth of 12000 and 1000 iterations, using *avgdist()* in the *vegan* package and custom functions in R [51,60]. To construct the phylogenic tree, we followed the steps outlined in the *Phylogenetic reconstruction of tick-borne pathogens and R. buchneri* sections, except for the bootstrapping step. The final tree was imported into the *phyloseq* object, and both weighted and unweighted UniFrac were performed within *phyloseq v 1.50.0* [56]. Statistical analyses of the distance matrices were performed using PERMANOVA from the *adonis2* function to examine the additive effects of host, damage status, engorgement status, and date of sample collected [60]. To visualize the distance matrices in multivariate ordination, we used Principal Co-ordinate Analysis (PCoA) and Non-metric Multidimensional Scaling (NMDS) and plotted the results using *ggplot2* v.4.0.2 [61].

iii) Phylogenetic reconstruction of tick-borne pathogens and *R. buchneri*

Phylogenetic reconstruction of ASVs assigned as *Borrelia burgdorferi*, *Anaplasma phagocytophilum*, and *R. buchneri* endosymbiont was performed as described previously, with modifications as briefed here. Initially, we downloaded 16S V4 regions of *Borrelia burgdorferi sensu lato complex*, *Borrelia miyamotoi*, *Anaplasma* spp., and *Rickettsia* spp. from NCBI Genbank and RefSeq, aligned the ASVs with these sequences using DECIPHER v.3.2.0 [64]. Optimized phylogenies were generated using the phangorn package v.2.12.1 [65], and for each phylogeny generated Table 1, a maximum-likelihood tree was generated using the distance-based method, and the best substitution model was identified using phangorn v.2.12.1 [65]. The branch confidence of each of the final phylogenetic reconstructions was assessed using 1,000 bootstrap replicates, and the final phylogenetic trees were visualized using ggtree v.4.3.0 and ggtreeExtra v.1.16 [66,67].

**Table 1:** Biosamples^a^.

• SAMN61871380 : T001 (TaxID: 1595979)
• SAMN61871381 : T002 (TaxID: 1595979)
• SAMN61871382 : T003 (TaxID: 1595979)
• SAMN61871383 : T004 (TaxID: 1595979)
• SAMN61871384 : T005 (TaxID: 1595979)
• SAMN61871385 : T006 (TaxID: 1595979)
• SAMN61871386 : T007 (TaxID: 1595979)
• SAMN61871387 : T008 (TaxID: 1595979)
• SAMN61871388 : T009 (TaxID: 1595979)
• SAMN61871389 : T010 (TaxID: 1595979)
• SAMN61871390 : T012 (TaxID: 1595979)
• SAMN61871391 : T013 (TaxID: 1595979)
• SAMN61871392 : T014 (TaxID: 1595979)
• SAMN61871393 : T015 (TaxID: 1595979)
• SAMN61871394 : T016 (TaxID: 1595979)
• SAMN61871395 : T017 (TaxID: 1595979)
• SAMN61871396 : T018 (TaxID: 1595979)
• SAMN61871397 : T019 (TaxID: 1595979)
• SAMN61871398 : T020 (TaxID: 1595979)
• SAMN61871399 : T021 (TaxID: 1595979)
• SAMN61871400 : T023 (TaxID: 1595979)
• SAMN61871401 : T024 (TaxID: 1595979)
• SAMN61871402 : T025 (TaxID: 1595979)
• SAMN61871403 : T026 (TaxID: 1595979)
• SAMN61871404 : T027 (TaxID: 1595979)
• SAMN61871405 : T028 (TaxID: 1595979)
• SAMN61871406 : T029 (TaxID: 1595979)
• SAMN61871407 : T030 (TaxID: 1595979)
• SAMN61871408 : T031 (TaxID: 1595979)
• SAMN61871409 : T032 (TaxID: 1595979)
• SAMN61871410 : T033 (TaxID: 1595979)
• SAMN61871411 : T034 (TaxID: 1595979)
• SAMN61871412 : T035 (TaxID: 1595979)
• SAMN61871413 : T036 (TaxID: 1595979)
• SAMN61871414 : T037 (TaxID: 1595979)
• SAMN61871415 : T038 (TaxID: 1595979)
• SAMN61871416 : T039 (TaxID: 1595979)
• SAMN61871417 : T040 (TaxID: 1595979)
• SAMN61871418 : T041 (TaxID: 1595979)
• SAMN61871419 : T043 (TaxID: 1595979)
• SAMN61871420 : T044 (TaxID: 1595979)
• SAMN61871421 : T045 (TaxID: 1595979)
• SAMN61871422 : T046 (TaxID: 1595979)
• SAMN61871423 : T047 (TaxID: 1595979)
• SAMN61871424 : T048 (TaxID: 1595979)
• SAMN61871425 : T049 (TaxID: 1595979)
• SAMN61871426 : T050 (TaxID: 1595979)
• SAMN61871427 : T051 (TaxID: 1595979)
• SAMN61871428 : T052 (TaxID: 1595979)
• SAMN61871429 : T053 (TaxID: 1595979)
• SAMN61871430 : T054 (TaxID: 1595979)
• SAMN61871431 : T055 (TaxID: 1595979)
• SAMN61871432 : T056 (TaxID: 1595979)
• SAMN61871433 : T057 (TaxID: 1595979)
• SAMN61871434 : T059 (TaxID: 1595979)
• SAMN61871435 : T060 (TaxID: 1595979)
• SAMN61871436 : T061 (TaxID: 1595979)
• SAMN61871437 : T062 (TaxID: 1595979)
• SAMN61871438 : T063 (TaxID: 1595979)
• SAMN61871439 : T064 (TaxID: 1595979)
• SAMN61871440 : T065 (TaxID: 1595979)
• SAMN61871441 : T066 (TaxID: 1595979)
• SAMN61871442 : T067 (TaxID: 1595979)
• SAMN61871443 : ext_negative_1 (TaxID: 256318)
• SAMN61871444 : ext_negative_2 (TaxID: 256318)
• SAMN61871445 : ext_negative_3 (TaxID: 256318)
• SAMN61871446 : PCR_1_negative (TaxID: 256318)
• SAMN61871447 : PCR2_-ve_1 (TaxID: 256318)
• SAMN61871448 : PCR2_-ve_2 (TaxID: 256318)
• SAMN61871449 : PCR3_-ve_1 (TaxID: 256318)
Raw sequences (SRA):
• SRR39769114
• SRR39769115
• SRR39769119
• SRR39769118
• SRR39769120
• SRR39769121
• SRR39769122
• SRR39769123
• SRR39769102
• SRR39769104
• SRR39769105
• SRR39769106
• SRR39769107
• SRR39769111
• SRR39769108
• SRR39769109
• SRR39769110
• SRR39769112
• SRR39769113
• SRR39769101
• SRR39769100
• SRR39769103
• SRR39769116
• SRR39769117
• SRR39769099
• SRR39769098
• SRR39769097
• SRR39769096
• SRR39769095
• SRR39769092
• SRR39769091
• SRR39769090
• SRR39769089
• SRR39769088
• SRR39769087
• SRR39769086
• SRR39769085
• SRR39769084
• SRR39769083
• SRR39769075
• SRR39769073
• SRR39769072
• SRR39769071
• SRR39769070
• SRR39769069
• SRR39769068
• SRR39769066
• SRR39769065
• SRR39769064
• SRR39769094
• SRR39769093
• SRR39769061
• SRR39769060
• SRR39769058
• SRR39769057
• SRR39769056
• SRR39769059
• SRR39769055
• SRR39769054
• SRR39769082
• SRR39769081
• SRR39769079
• SRR39769078
• SRR39769077
• SRR39769080
• SRR39769076
• SRR39769074
• SRR39769067
• SRR39769063
• SRR39769062

^*a*^ the raw sequencing data and associated meta-data are on NCBI: Bioproject: PRJNA1499239

iv) Pathogen co-occurrence analysis:

To evaluate the relationship between *B. burgdorferi* and *A. phagocytophilum*, a summary figure (Fig 1), was constructed containing total counts of ASVs assigned to *B. burgdorferi* and *A. phagocytophilum*.

**Fig 1:**
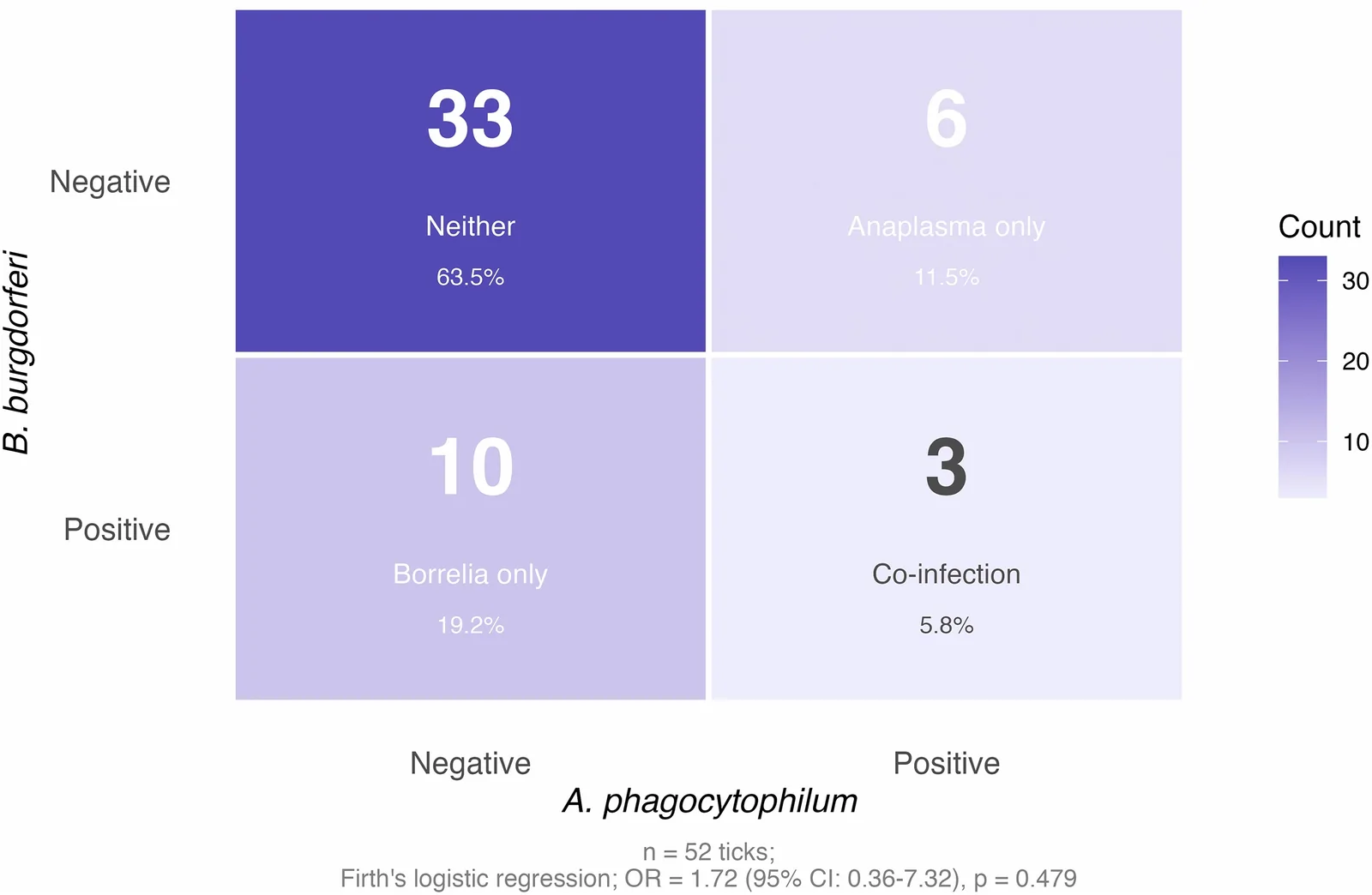
Co-occurrence matrix of *B. burgdorferi* and *A. phagocytophilum* from *I. scapularis* ticks collected from CD-NHAs. Contingency matrix of detection outcomes across *I. scapularis* screened (n = 52). Most ticks were negative for both pathogens (33/52, 63.5%), while 10 (19.2%) carried *B. burgdorferi* alone, 6 (11.5%) carried *A. phagocytophilum* alone, and 3 (5.8%) were co-infected. Firth’s penalized logistic regression found no evidence of association between the two pathogens (OR = 1.72, 95% CI 0.36–7.32, p = 0.479).

A 2 × 2 contingency matrix was generated to visualize co-occurrence patterns using ggplot2 v.4.2.0 [61]. The statistical association was assessed using Firth’s penalized likelihood logistic regression using *logistf* v.1.26.1 [68] with *B. burgdorferi* presence as the response variable and *A. phagocytophilum* as the predictor variable. This method was selected over standard logistic regression because of the smaller sample size and low cell counts. To determine if host-specific factors influenced this association, a secondary Firth’s model was used with host species included as an additive covariate. Odds ratios and 95% confidence intervals were used to quantify the strength and direction of the association between the pathogens. Please see Table 2 for the pathogen abundance and total abundance count for each tick that was associated with one or more pathogens.

**Table 2:** Tick descriptions and associated pathogens.

Sample-name	Tick description^a^	Anaplasma ASV 295	Anaplasma ASV 296	Anaplasma ASV 297	Borrelia ASV 912
T014	M/IxS	0 (N)	0 (N)	9 (Y)	0 (N)
T015	F/IxS	147 (Y)	0 (N)	22563 (Y)	0 (N)
T026	F/IxS	0 (N)	0 (N)	28 (Y)	206 (Y)
T030	M/IxS	0 (N)	0 (N)	8 (Y)	0 (N)
T034	F/IxS	0 (N)	0 (N)	3 (Y)	0 (N)
T035	F/IxS	0 (N)	0 (N)	10 (Y)	0 (N)
T037	F/Ix?	0 (N)	47 (Y)	276 (Y)	109 (Y)
T044	F/Ix?	0 (N)	0 (N)	918 (Y)	0 (N)
T061	F/IxS	0 (N)	0 (N)	14 (Y)	2495 (Y)
T007	F/IxS	0 (N)	0 (N)	0 (N)	2188 (Y)
T020	F/IxS	0 (N)	0 (N)	0 (N)	6 (Y)
T032	F/IxS	0 (N)	0 (N)	0 (N)	469 (Y)
T036	F/IxS	0 (N)	0 (N)	0 (N)	10 (Y)
T043	F/IxS	0 (N)	0 (N)	0 (N)	146 (Y)
T045	F/Ix?	0 (N)	0 (N)	0 (N)	188 (Y)
T057	F/IxS	0 (N)	0 (N)	0 (N)	236 (Y)
T059	F/IxS	0 (N)	0 (N)	0 (N)	152 (Y)
T063	F/IxS	0 (N)	0 (N)	0 (N)	27 (Y)
T066	F/Ix?	0 (N)	0 (N)	0 (N)	18 (Y)

^a^Legend for Tick Description: M (Male) F (Female) Ix (Ixodes) S (Scapularis)? (unknown)

## Results

### Sample and sequencing overview

A total of 63 ticks were collected from the CD-NHAs animals and a further 4 unattached ticks were collected in Eastern Ontario between May 19 and September 27, 2023 (bovine: n = 35; equine: n = 22; canine: n = 6; unattached: n = 4). The unattached ticks were found walking on the CD-NHAs. Morphological identification revealed 55 *Ixodes scapularis*, 11 *Ixodes* spp., and 1 *Dermacentor variabilis*. Out of 67 ticks, 61 were identified as adult females, and six were identified as adult males. The physical status of the ticks, such as engorgement and damage, was also categorized. 32 ticks (49.2%) were identified as fully engorged, while 33 host-attached ticks were classified as either partially engorged or non-engorged, and the remaining two were non-engorged. 18 ticks were classified as damaged due to missing hypostome or incomplete morphological structure, while the remaining 49 ticks were deemed intact.

Total genomic DNA was extracted from 67 samples. However, seven samples failed to amplify during the library preparation and were excluded from the analysis. The remaining 60 samples were sequenced alongside three no-template controls for DNA extraction and four no-template controls for PCR, yielding a total of 14682960 paired-end reads. (mean ± SD: 127,587 ± 117,697 reads per sample; range: 128–562,142). The negative controls collectively contributed 903,102 paired-end reads. After primer trimming with cutadapt (minimum Phred score: 20; minimum length: 50 bp), 14,608,274 paired-end reads were retained. Following denoising, merging, and chimera removal in DADA2, 2005 ASVs were yielded. After contaminant ASV removal using *decontam* and basic quality removal, 52 samples (equine-attached ticks (n = 27), bovine-attached ticks (n = 18), canine-attached ticks (n = 4), and unattached ticks (n = 3)) with 882 ASVs were retained for downstream analyses. All of the analyzed ticks were identified as *Ixodes species*.

### Pathogen summary

As shown in Fig 1, of the 52 samples retained for downstream analysis, 19 (36.5%) tested positive for at least one tick-borne pathogen. Fig 1 is a co-occurrence matrix of *B. burgdorferi* and *A. phagocytophilum* from *I. scapularis* ticks collected from CD-NHAs**.** Contingency matrix of detection outcomes across *I. scapularis* screened (n = 52). Most ticks were negative for both pathogens (33/52, 63.5%), while 10 (19.2%) carried *B. burgdorferi* alone, 6 (11.5%) carried *A. phagocytophilum* alone, and 3 (5.8%; 15.7% of pathogen-positive samples) were co-infected. Firth’s penalized logistic regression found no evidence of association between the two pathogens (OR = 1.72, 95% CI 0.36–7.32, p = 0.479).. Furthermore, the pathogen’s presence varied by host attachment. Equine-attached ticks had the highest proportion of pathogen-positive samples, with 15 of 27 ticks positive for at least one tick-borne pathogen (55.5%), followed by canine-attached ticks with 1 of 4 positive samples (25.0%) and bovine-attached ticks with 3 of 18 positive samples (16.7%). No tick-borne pathogens were detected in the unattached ticks. Co-infections were most frequent in equine-attached ticks, where 3 of 27 samples showed co-detection (11.1%); no co-infections were detected in canine-attached, bovine-attached, or unattached ticks. To evaluate the co-occurrence of *Borrelia* spp. and *Anaplasma* spp., we modeled *Borrelia* spp. detection as a function of *Anaplasma* spp. detection using Firth’s penalized logistic regression, accounting for host attachment, engorgement status, and damage status. *Anaplasma* spp., co-detection was associated with a 1.71-fold increase in the odds of *Borrelia* detection (OR = 1.71, 95% CI: 0.357–7.316, p = 0.479), though this did not reach statistical significance. Overall, these suggest that pathogen detection varied among host attachment, and the observed co-occurrence pattern between *Borrelia* spp. and *Anaplasma* spp. was not strong enough to support a statistically significant association.

### *I. scapularis* bacterial community structure still dominated by *R. buchneri*

The bacterial communities in tick samples at the phylum level were dominated by Pseudomonodota, although their relative abundances varied among samples. Actinomycetota and Bacillota I were also consistently detected and contributed a large proportion of the community. Phyla such as Bacillota_A_368345, Bacteroidota, Planctomycota, Chloroflexota, and 15 other phyla were detected in low abundance. At the genus level, *Rickettsia* was the dominant genus across most samples. Mean relative abundance of *Rickettsia* was highest in ticks attached to equine hosts (55.2% ± 23.9%), followed by canine-attached ticks (52.5% ± 21.0%) and bovine-attached ticks (37.8% ± 28.4%). Unattached ticks had a lower mean relative abundance of *Rickettsia* (11.7% ± 16.5%) and comparatively higher relative abundances of *Staphylococcus*, *Lawsonella*, and *Corynebacterium*. When stratified by engorgement status, *Rickettsia* remained the dominant genus in both engorged and partially engorged ticks. *Staphylococcus* was the second most abundant genus in both groups (non-engorged: 17.5% ± 9.5%; engorged: 18.7% ± 12.3%), followed by *Cutibacterium* (non-engorged: 11.1% ± 5.3%; engorged: 18.3% ± 12.1%). *Paracoccus* was more abundant in engorged ticks (3.2% ± 4.0%) compared to non-engorged ticks (1.0% ± 1.2%), though this difference showed high variability. Overall, these patterns indicate that *Rickettsia* remained a major component of the *I. scapularis* bacterial community across host and engorgement groups, suggesting that the *R. buchneri*-specific blocking primer reduced but did not eliminate *Rickettsia* 16S rRNA sequences during library preparation. Fig 2 is a Stacked barplot that shows the relative abundance of 20 most abundant genera in each of the tick samples with all remaining taxa collapsed under “Other” faceted by A) engorgement status and B) host of collection or unattached.

**Fig 2:**
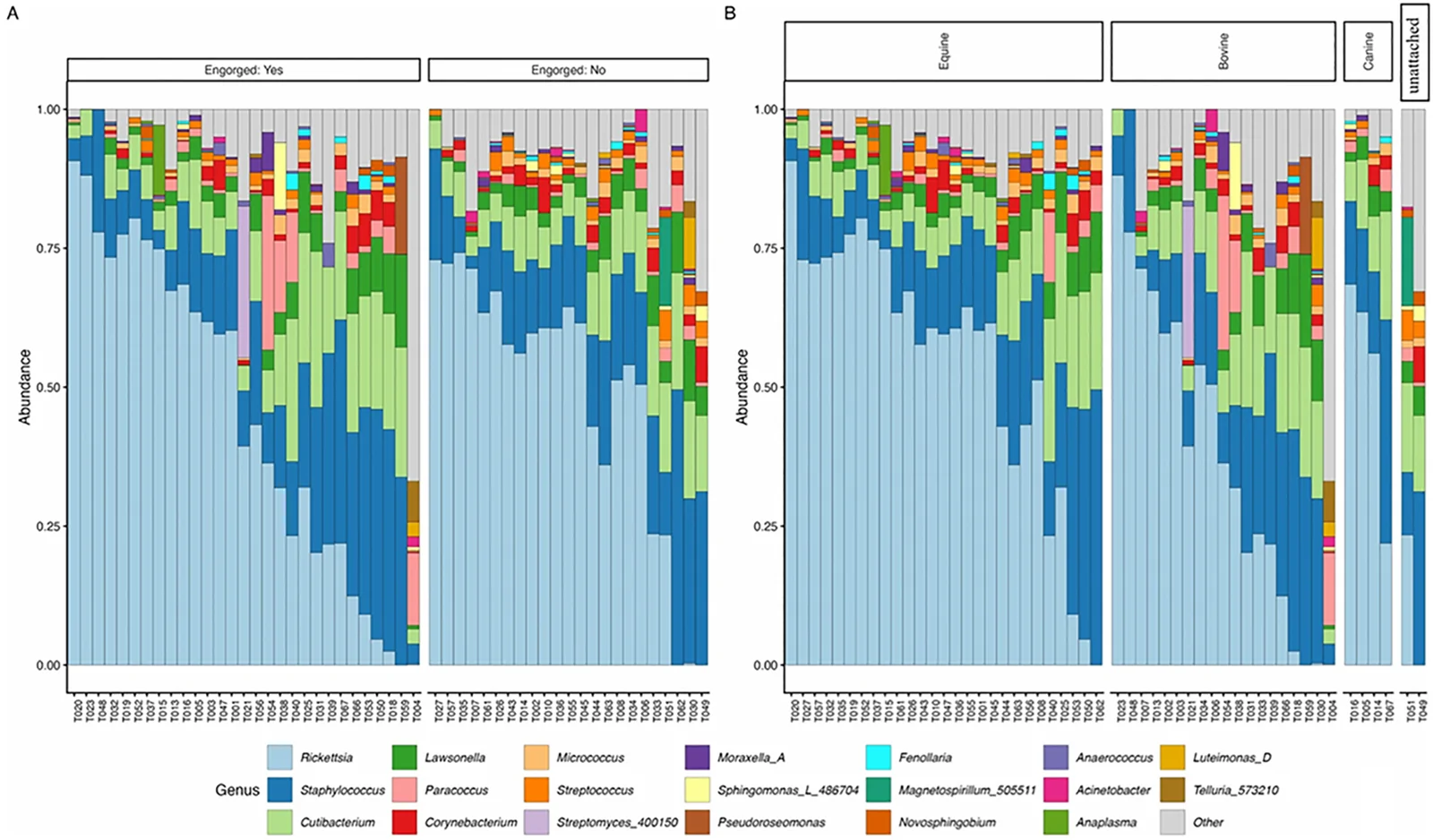
Genus-level taxonomic composition of ticks collected from CD-NHAs. Stacked barplot shows the relative abundance of 20 most abundant genera in each tick samples with all remaining taxa collapsed under “Other” faceted by A) engorgement status and B) host of collection or unattached.

### *I. scapularis* bacterial diversity depends on the presence of *Rickettsia* ASVs

We estimated several alpha-diversity indices for each sample, but we mainly focused on the Hill numbers. with q = 0 representing observed richness, q = 1 representing the exponential of Shannon entropy (Shannon – Hill), and q = 2 representing inverse Simpson diversity (Simpson-Hill). Observed richness (when q = 0) was statistically insignificant and smaller effect sizes irrespective of the host attachment (R^2^ = 0.058; p = 0.91), engorgement (R^2^ = 0.0031; p = 0.99), and damage status (R^2^ = 0.14; p = 0.33), indicating limited variation in taxon richness across these sample categories. In contrast, Shannon – Hill (when q = 1) differed significantly among host groups and showed a large effect size (Kruskal-Wallis: R² = 0.18, p = 0.014). Unattached ticks had the highest mean Shannon-Hill diversity, whereas equine-attached ticks had the lowest mean diversity. Pairwise comparisons showed significant differences between equine- and bovine-attached ticks (adjusted p = 0.041) and between unattached and equine-attached ticks (adjusted p = 0.041). Shannon-Hill diversity was not significantly associated with engorgement status (R² = 0.059, p = 0.7) or damage status (R² = 0.13, p = 0.36). However, Simpson-Hill diversity (q = 2) differed significantly among host groups, with a comparable effect size (R² = 0.18, p = 0.014). Dunn’s post hoc test indicated significant differences between equine- and bovine-attached ticks (adjusted p = 0.041) and between equine-attached and unattached ticks (adjusted p = 0.041). Simpson-Hill diversity was not significantly associated with either engorgement or damage status. Overall, these results indicate that host attachment, rather than engorgement or damage status, was the main factor associated with variation, while observed richness remained similar across categories.

Multivariate community structure was assessed using UniFrac and Bray–Curtis distance matrices. Unweighted UniFrac ordination showed no significant clustering by host group, damage status, engorgement status, or sampling date. Host group explained only a small proportion of variation in unweighted UniFrac distances (PERMANOVA: F = 1.0217, R² = 0.0695, p = 0.399), as did damage status (F = 0.8046, R² = 0.01824, p = 0.783) and engorgement status (F = 0.8989, R² = 0.02038, p = 0.578). Sampling date explained a larger proportion of the variation (R² = 0.37032), but this effect was not significant (F = 0.9073, p = 0.909), likely reflecting the high number of date categories relative to the sample size. In contrast, abundance-weighted distance metrics showed a weak trend toward host attachment. Weighted UniFrac showed a marginal, non-significant trend for host attachment (PERMANOVA: F = 1.9403, R² = 0.11949, p = 0.088), while damage status, engorgement status, and sampling date remained non-significant. Homogeneity of multivariate dispersion did not differ significantly among host groups for weighted UniFrac distances (PERMDISP: F = 1.27, p = 0.267), indicating that the weak trend of host attachment was not driven by within-group dispersion. A similar non-significant host-attached trend was observed using Bray–Curtis dissimilarity (PERMANOVA: F = 1.8212, R² = 0.11274, p = 0.076), with no significant difference in dispersion among host attachment (PERMDISP: F = 1.45, p = 0.245). Together, these results indicate that abundance-unweighted community structure showed no evidence of grouping by host or tick condition, whereas abundance-weighted metrics suggested a weak but non-significant trend toward a host-attached pattern.

Because *Rickettsia*-assigned ASVs remained dominant despite the use of blocking primers, ASVs assigned to *Rickettsia* spp. were removed, and alpha-diversity analyses were repeated to assess patterns in the non-rickettsial bacterial community. After *Rickettsia* removal, observed richness (q = 0) was not significantly associated with host attachment (R² = 0.027, p = 0.60), damage status (R² = 0.0056, p = 0.98), or engorgement status (R² = 0.14, p = 0.36), consistent with patterns observed in the full dataset. In contrast to the full dataset, Shannon-Hill diversity (q = 1) was no longer significantly associated with any of the tested variables and exhibited low effect sizes. Simpson-Hill diversity (q = 2), however, remained significantly associated with host attachment, with a large effect size (R² = 0.16, p = 0.024). Post hoc pairwise comparisons did not identify significant differences among host groups after correction, although weak trends were observed between canine-attached and unattached ticks, equine-attached and unattached ticks, and canine- and equine-attached ticks (adjusted p = 0.077). Damage status and engorgement status were not significantly associated with Simpson-Hill diversity. Furthermore, the multivariate ordination yielded results like those previously observed for unweighted UniFrac; however, both weighted UniFrac and Bray-Curtis showed a weak, insignificant trend for Damage status, whereas host attachment, engorgement status, and date of sampling remained insignificant. Overall, the removal of *Rickettsia* ASVs reduced the host-associated alpha-diversity signal observed in the full dataset and suggested that residual variation in the non-*Rickettsia* community was more weakly associated with tick-damage status than with host attachment.

### Phylogenetic reconstruction of *I. scapularis*-associated pathogens and endosymbiont

Phylogenetic reconstruction was performed for ASVs assigned to *Borrelia* spp. (n = 1), *Anaplasma* spp. (n = 2), and *Rickettsia* spp. (n = 16). Sequence alignment showed that one *Rickettsia*-assigned ASV, ASV1, shared 100% identity across the 16S rRNA V4 region with *R. buchneri* (NR_134842.2), *R. monacensis* (NR_115686.1), and *R. monteiroi* (NR_133040.1). The remaining 15 *Rickettsia*-assigned ASVs shared >99% sequence identity with multiple *Rickettsia* reference sequences, indicating that these ASVs could be confidently assigned to the genus Rickettsia but could not be unambiguously resolved to species using the V4 region alone. The single *Borrelia*-assigned ASV, ASV46, shared 100% sequence identity with *Borreliella burgdorferi* reference sequence MH781147.1 and *Borreliella bissettiae* NR_148750.1, supporting its placement within the *B. burgdorferi* sensu lato complex rather than allowing definitive species-level assignment. Similarly, *Anaplasma*-assigned ASV13 shared 100% sequence identity with the *A. phagocytophilum* reference sequences NR_044762.1 and HG916766.1, whereas ASV940 clustered within the *A. phagocytophilum* clade. Both ASVs were distinct from *A. odocoilei* and *A. marginale*, supporting their placement within the *A. phagocytophilum*. Overall, these phylogenetic results support genus-level detection of *Rickettsia*, *Borreliella*, and *Anaplasma* ASVs, while highlighting the limited species-level resolution of the 16S rRNA V4 region for *I. scapularis* endosymbionts and pathogens.

## Discussion

The integration of One-Health-guided sentinel surveillance plays a critical role in predicting the spread of zoonotic vector-borne diseases driven by anthropogenic climate change [9,69]. In this study, we tested the sentinel model by repeated sampling of ticks from CD-NDAs at a farm sanctuary in Eastern Ontario. By characterizing the local tick population and applying High-Throughput Sequencing (HTS) to detect bacterial tick-borne pathogens, this study suggests that tick collection based on CD-NHAs may also serve as a useful means of identifying emerging and established vector-borne pathogen risks, as previously reported for other domestic and companion animals. To investigate bacterial pathogens in Eastern Ontario, we targeted the V4 hypervariable region of the 16S rRNA gene. This broad amplicon-based approach enabled the detection of reads assigned to the *B. burgdorferi sensu lato* complex, *A. phagocytophilum*, and even *Babesia* sp., demonstrating the potential of HTS to screen for multiple bacterial pathogen-associated taxa in a single assay, but it lacks the sensitivity of qPCR [30]. This limitation of HTS microbiome profiling in *I. scapularis* is mainly due to the dominance *of R. buchneri*, a maternally inherited endosymbiont that can overwhelm bacterial community profiles and reduce sensitivity in detecting low-abundance taxa. To address this technical constraint, we used a *R. buchneri*-specific blocking primer to suppress amplification of endosymbionts and improve recovery of the non-rickettsial bacterial community.

Sentinel-based surveillance of ticks, incorporating morphometrics and HTS, identified that most ticks collected from CD-NHAs were *I. scapularis*, whereas 17% could only be resolved to the genus level as Ixodes spp., due to engorgement or missing structures. This finding suggests CD-NDAs are consistently exposed to Ixodes spp., a primary vector for diverse microbial pathogens, including eukaryotic and viral pathogens [26,70–72]. 16S amplicon sequencing, paired with a blocking primer, detected an overall bacterial pathogen prevalence of 36.5% (19/52 ticks), with one *B. burgdorferi*-associated ASV at 19.2% (10/52 ticks), and two *A. phagocytophilum*-associated ASVs of the prevalence of 11.5% (6/52 ticks). Although co-infection was detected in 5.7% (3/52) of ticks, the association between *Borrelia* spp. and *Anaplasma* spp. did not reach statistical significance, likely due to limited power given the small number of co-infected samples. However, previous studies have reported a higher prevalence of co-infection with *Anaplasma* spp. and *Borrelia* spp., with an underlying mechanism involving immunomodulation of IAFGP, an antifreeze glycoprotein, by *A. phagocytophilum*, which alters biofilm formation, reducing the complexity and robustness of the commensal microbial network and ultimately making I. scapularis susceptible to invasions by other pathogens [73–76]. Furthermore, the *Borrelia* prevalence observed in our study was lower than the provincial active/passive surveillance estimate for *B. burgdorferi* (28.8%), whereas the *Anaplasma*-associated prevalence was higher than the reported provincial estimate of 2.9%, suggesting that infection prevalence may depend on the microhabitat [77–79]. In the context of ongoing tick range expansion in Canada and Ontario, sentinel-based sampling among community dwelling animals such as those living in farm animal sanctuaries can help identify emerging tick populations and local hotspots for established or novel tick-borne pathogens by complementing conventional passive surveillance and active drag sampling, capturing tick exposure in spaces where humans, domestic animals, and wildlife overlap, supporting the identification of emerging tick populations and local hotspots for established or novel tick-borne pathogens within a One Health framework [80,81].

In addition to tick-borne pathogens, bacterial community structure was still dominated by 16 *Rickettsia*-assigned ASVs across the host attachment, engorgement, and damage statuses. Phylogenetic reconstruction confirmed that the most abundant ASV (i.e., ASV1) shared 100% sequence identity with *R. buchneri* (NR_134842.2), *R. monacensis* (NR_115686.1), and *R. monteiroi* (NR_133040.1), and the remaining 15 ASVs shared > 99% sequence identity with the same reference. While the blocking primer suppressed most rickettsial reads in unattached ticks (mean relative abundance: 11.65 ± 16.5%), it did not suppress them efficiently in engorged ticks (mean relative abundance: 45.4% ± 29.6%) or partially engorged ticks (mean relative abundance: [41].8% ± 23.7%). This pattern may reflect differences in starting endosymbiont load among tick physiological states. Previous studies have shown that the *R. buchneri* load increases during blood-feeding and dominates the bacterial community of adult female *I. scapularis*, particularly in reproductive tissues, where it is hypothesized to supplement the tick’s nutrient-deficient blood meal diet by synthesizing biotin and folate, which are deficient in vertebrate blood, and its abundance increases markedly in association with oviposition [82–84]. As the blocking primer was originally developed and optimized for non-engorged adult female *I. scapularis*, which had comparatively lower rickettsial abundance (~ 50%), its performance may have been reduced in samples with much higher endosymbiont template concentrations. Thus, residual *Rickettsia* amplification in engorged and partially engorged ticks likely reflects both the biological dominance of *R. buchneri* and a technical limitation of blocking-primer efficiency when the target template is highly abundant. Despite this limitation, the primer sufficiently reduced endosymbiont masking to provide critical insights into the underlying non-rickettsial bacterial community of *I. scapularis*, which was characterized by *Staphylococcus*, *Cutibacterium*, *Lawsonella, Corynebacterium, Moraxella A*, *Micrococcus*, and *Paracoccus*, all of which are consistently reported in *I. scapularis* as transient/facultative microbes that were acquired horizontally during blood feeding (host-derived) or off-host (environmentally-derived) [41,75,85,86]. These transient microbes, even at low abundance, might contribute to activation of the tick immune system by triggering a DUOX-mediated immune response, which upregulates reactive oxygen species (ROS) and leads to the expression of antimicrobial peptides (AMPs) [41,87]. At the same time, transient microbes such as *Staphylococcus* and *Corynebacterium* are known to be targeted by *dae2*, a bactericidal effector horizontally acquired *by I. scapularis* approximately 40 Mya, which is secreted into the tick gut and saliva during feeding [75,88,89]. Both immune responses are complementary and might contribute to colonization resistance in the tick gut against pathogens. These findings highlight that transient microbes actively engage the tick’s immune system and may indirectly shape its vector competence, reinforcing the use of a blocking primer to suppress endosymbionts and reveal ecologically and functionally relevant microbial diversity that would otherwise remain masked in 16S-based amplicon studies.

Additionally, transient microbes also play a crucial role in shaping the microbial community of *I. scapularis*, as most are acquired either during the off-host unattached period or while feeding on the host. As a result, the host’s blood and skin microbiome becomes a key determinant of the microbial diversity and structure in ticks [85]. Bacterial alpha diversity, assessed using Hill numbers, showed that host attachment primarily drove variation in the bacterial community. Specifically, Shannon-Hill and Simpson Hill indices differ significantly among host attachments, with equine-attached ticks showing the lowest mean richness and evenness, whereas unattached ticks exhibit the highest. This result aligns with previous studies demonstrating the impact of the host-blood meal on the microbial structure of ticks, and can be extended to CD-NHA hosts, which have been studied sparsely in the context of *I. scapularis* microbial structure [22,90]. Notably, the unattached ticks had the highest richness, consistent with exposure to diverse soil- and environment-associated microbes while off-host. However, further experimental validation of this hypothesis is warranted, given the current study’s low sample size per host. Conversely, the observed richness was not significantly associated with any variables, suggesting the number of taxa present in *I. scapularis* remained similar irrespective of the host attachment, while the community composition of those taxa differed. This dissociation between richness and evenness is consistent with previous *I. scapularis* microbial diversity studies, which showed that the mean Simpson diversity index was negatively correlated with bacterial load, which is dominated mainly by *R. buchneri*, especially with massive proliferation during feeding and oviposition [84]. This interpretation is also supported by the transient influence of blood meal and the natural environment on tick bacterial communities, with host-associated transient taxa detectable shortly after feeding but declining over time, suggesting that their contribution to observed richness is ephemeral and highly variable among individual ticks rather than reflecting a stable internal microbial community [22]. While alpha diversity indices were used to capture the differences in within-sample richness and evenness in bacterial communities, beta-diversity was used to investigate the community composition between the samples, where unweighted UniFrac showed no significant clustering by host attachment, engorgement, damage, or sampling date, suggesting the presence or absence of bacterial taxa was similar across variables. In contrast, abundance-weighted analyses (UniFrac and Bray-Curtis) showed a non-significant trend for the host-attached group, suggesting that the taxonomic composition remained the same, whereas the relative abundances of taxa differed by host association, consistent with previous studies [90,91]. The statistical insignificance in our study likely reflects a limited sample size per group and high within-group dispersion, the latter confirmed to be non-significant by PERMDISP, ruling out heterogeneity of variance as a confounding factor. Following the removal of *Rickettsia* ASVs, the host-associated significance for Shannon-Hill was eliminated, whereas the significance for Simpson-Hill was retained, suggesting that the small number of dominant non-rickettsial bacteria, likely transient microbes acquired from the host skin microbiome, varied in abundance across host groups, which is consistent with the tick immune dynamics on the transient microbes. Beta diversity after the removal of *Rickettsia* showed a weak, non-significant trend for damage status in weighted matrices (Weighted UniFrac and Bray-Curtis), suggesting that the absence of the hypostome might affect the abundance of transient microbes due to methodological variability, as damaged ticks may have increased exposure to chemical reagents during the DNA extraction and library preparation. Given the limited number of samples, this observation warrants further investigation, specifically assessing the impact of the tick’s structural integrity on microbial composition. Collectively, these findings suggest that host blood meal and skin microbiome shape the *I. scapularis* bacterial community structure, while tick engorgement or damage has little influence on community diversity.

Beyond taxonomic and community structures, the phylogenetic reconstruction of the tick-borne pathogens and endosymbiotic ASVs detected in our study revealed a main limitation of 16S rRNA V4 amplicon sequencing, the inability to achieve species-level resolution. For instance, the dominant *Rickettsia*-assigned ASV (i.e., ASV1) shared 100% sequence identity and placed them closely with *R. buchneri* (NR_134842.2), *R. monacensis* (NR_115686.1), and *R. monteiroi* (NR_133040.1). Previous phylogenomic analyses on various rickettsial species using single-copy core orthologs clearly distinguished *R. monacensis* IrR/Munich (*I. ricinus*), *Rickettsia* endosymbiont of *I. pacificus* (*I. pacificus*), *R. buchneri* strain ISO7 (*I. scapularis*), and *R. buchneri strain* Wikel (*I. scapularis*), with a distinct monophyletic lineage, which suggests 16S–V4 amplicon may not be appropriate for the identification of rickettsial species [91]. While the ecological context of *I. scapularis* from Canada strongly supports its identification as *R. buchneri* [30], the co-identified *R. monacensis*, a spotted fever group (SFG) rickettsial species, is mainly associated with *I. ricinus* in European countries and has not been reported in the I. scapularis microbiome of the Canadian tick population [92,93]. Similarly, *Borreliella*-assigned ASV also shared 100% sequence identity with both *B. burgdorferi* (MH781147.1), which is identified as a dominant genospecies within the *B. burgdorferi* sensu lato complex across Canada, and *B. bissettiae* (NR_148750.1), placing the identified ASV into *B. burgdorferi sensu lato* complex. Experimental studies have demonstrated I. scapularis nymphs have lower transmission competence for *B. bissetiae* compared to *B. burgdorferi sensu stricto*; however, *I. scapularis* could still acquire it [94]. Hence, the identification of *Borrelia spp*. has important implications for public health, as the clinical manifestations and diagnostic methods differ among *Borrelia* genospecies. Even though both the *Anaplasma*-assigned ASVs had placement within *A. phagocytophilum* clade, it was still not able to distinguish between the human-pathogen variant (Ap-ha) and the non-pathogen variant (Ap-var1). Currently, in North American regions, I. scapularis acts as a vector for both variants, with a higher incidence of human granulocytic anaplasmosis associated with Ap-ha than with Ap-var1 [95]. Hence, the distinction between strains is directly relevant to public risk assessment. Collectively, these results suggest that 16S rRNA V4 regions can be a valuable tool for genus-level taxonomic and community diversity assessments but have limitations in species- and strain-level resolution, which are required for public risk assessment of tick-borne pathogens.

## Conclusion

This study set out to investigate the prevalence of tick-borne diseases among CD-NHAs using a novel analysis technique.

In conclusion, this study demonstrates the potential of CD-NHAs for sentinel surveillance, complementing both active and passive tick surveillance frameworks within a One-Health context. By sampling ticks from a farm sanctuary, we captured vital data on tick-borne bacterial pathogens at the interconnected human-animal-environment interface, revealing that pathogen prevalence depends on the microhabitat structure. The analysis technique used here enabled us to characterize and detect both tick-borne pathogens, such as *B. burgdorferi sensu lato* and *A. phagocytophilum*, and the transient bacterial community, which plays a vital role in tick immunological landscapes. However, our study also highlighted a critical bottleneck in the 16S rRNA V4 amplicon region for taxonomic resolution, which limits our ability to distinguish pathogenic species and variants. Future tick surveillance incorporating a blocking primer must transition to long-read sequencing technologies that target the full-length 16S region. Coupling them with CD-NHA-based sentinel surveillance will provide robust data that will ultimately help predict, monitor, and mitigate established and emerging tick-borne disease threats in a changing climate.

Finally, demonstrating the practical value of sentinel studies such as this, the knowledge that several of the infected ticks collected in this study had been positive for *Anaplasma phagocytophilum* resulted in the speedy diagnosis of two of the equine participants when they demonstrated symptoms of lethargy and inappetence. They were immediately treated for Anaplasmosis and both made full recoveries. Tick borne diseases are common in this region, but frequently undiagnosed. It is hoped that, by reinforcing the awareness that ‘Lyme’ disease is not the only tick-borne threat to human and animal health will lead to the prevention of debilitating results from untreated tick-borne disease in both human and non-human animals.

## Supporting information

S1 File One Health genomic surveillance of ticks found on community-dwelling nonhuman ‘farm’ animals to identify microbes associated with tick-borne illnesses in Eastern Ontario. Details associated with using 16S rRNA V4 amplicon targeted sequencing (515F/906R) with custom Rickettsia buchneri blocking primers to boost sensitivity.
(PDF)
