# Supplementary material for: One Health genomic surveillance of ticks found on community-dwelling non-human ‘farm’ animals to identify microbes associated with tick borne illnesses in Eastern Ontario
Source: PLoS One. 2026 Oct 9;21(10):e0359215. doi: 10.1371/journal.pone.0359215 (PMC13652256; doi:10.1371/journal.pone.0359215)
Supplement: Supplementary file 1 [file pone.0359215.s001.pdf]

One Health genomic surveillance of ticks found on community-dwelling non-human ‘farm’ animals to identify microbes associated with tick-borne illnesses in Eastern Ontario

Sreevatshan K Srinivasan[1], Robert Colautti[1], Zhengxin Sun[1]

[1]Department of Biology, Queen's University

Sreevatshan K Srinivasan

Protocol Info:
Sreevatshan K Srinivasan, Robert Colautti, Zhengxin Sun . One Health genomic surveillance of ticks found on community-dwelling non-human ‘farm’ animals to identify microbes associated with tick-borne illnesses in Eastern Ontario. **protocols.io**
https://protocols.io/view/one-health-genomic-surveillance-of-ticks-found-on-j55pcq85p

Created: July 22, 2026

Last Modified: July 27, 2026

Protocol Integer ID: 321423

Funders Acknowledgements:
Robert Colautti
Grant ID: CIHR FRN 192227

Abstract

Companion animals, such as dogs and cats, and wildlife are recognized as sentinel species in One Health surveillance due to periodic exposure to ticks and infection by tick-borne pathogens (TBPs). Surveillance of this type is a strategy that captures a broad range of genetic, immunological, and behavioral factors that affect tick and disease dynamics. In contrast, non-human animals that are traditionally farmed for their meat and other products have been largely overlooked as sentinel species. Community-dwelling farm sanctuary residents, such as cattle, pigs, horses, and other traditionally ‘farmed’ non-human animals have close daily contact with human caregivers, making them excellent candidates for One Health surveillance. These nonhuman animals, often free-range in pastures and/or woodlands, bridging the territory inhabited by wildlife and that occupied by humans. This study collected over 60 ticks from four species of community-dwelling sanctuary residents (cattle, equines, canines, and felines) and screened bacteria for microbes associated with tick borne illnesses using 16S rRNA V4 amplicon targeted sequencing (515F/906R) with custom Rickettsia buchneri blocking primers to boost sensitivity. Almost one-third of ticks tested positive for potential pathogens, including approximately 20% for the causative agent of Lyme disease (Borrelia burgdorferi) and close to 10% for Anaplasma phagocytophilum, with a co-infection rate of around six percent. Utilizing community dwelling non-human sanctuary residents allowed us to detect potential threats to both non-human animal and human health and was instrumental in the rapid diagnosis of anaplasmosis in two a co-infection rate of around six percent. Utilizing community-dwelling non-human inappetence after being bitten by ticks.

Materials

Core Reagents

	A	B	C
	2× Q5 Mastermix	New England Biolabs	PCR Amplification
	SPRI magnetic beads	Prepared in-house following	PCR purification and size selection
	Nuclease-free water	NA	PCR setup, dilution, and elution
	80% ethanol	Prepared in-house	SPRI bead washing
	Just-a-Plate 96 PCR Purification and Normalization Kit	Charm Biotech, JN-120-10 or JN-120-2	Library normalization
	NEBNext Library Quant Kit for Illumina	NEB, E7630	qPCR library quantification
	PhiX Control v2	Illumina	Sequencing spike-in
	MiSeq reagent kit	v2 500-cycle	Paired-end sequencing

Consumables

	A	B
	1.5 mL microcentrifuge tubes	Eppendorf or equivalent
	Filtered pipette tips	P1000, P200, P20, and P10/P2
	PCR strip tubes	FroggaBio STF-A120 or equivalent
	Reagent reservoirs	Fisher Scientific 14-222-398 or equivalent
	96-well PCR normalization plates	Charm Biotech Just-a-Plate or equivalent
	PCR plate seals	Ultident semi-transparent polypropylene seal or equivalent
	qPCR plates	Bio-Rad HSP9601 or equivalent
	Optical qPCR sealing film	Bio-Rad MSB1001 or equivalent

A	B
0.5 mL thin-wall PCR tubes	For DeNovix assay, where used

Equipment

- Thermal cycler capable of holding at 10 °C.
- Magnetic rack suitable for 1.5 mL tubes.
- Microcentrifuge and plate centrifuge.
- Vortex mixer.
- Single-channel and multichannel pipettes.
- Repeater pipette, where available.
- Bluepippin and its Cassette with markers.
- Bio-Rad CFX96 or compatible qPCR instrument.
- Agarose gel electrophoresis equipment.
- Illumina MiSeq

Primer information

A	B	C
16S-27F-YMY	AGAGTTTGATYMTGGCTYAG	Full-length 16S forward primer
16S-1492R-HY	TACGGHTACCTTGTTAYGACTT	Full-length 16S reverse primer
18F-Rb-C3	Redacted	Rickettsia buchneri-specific blocking primer
515F	GTGYCAGCMGCCGCGGTAA	V4 forward primer
806R	GGACTACNVGGGTWTCTAAT	V4 reverse primer

Workflow overview

1

	Stage	Input	Description	Output
	1	Tick genomic DNA	Full-length 16S PCR with 27F-YMY, 1492R-HY, and 18F-Rb-C3	Purified full-length 16S amplicon, designated 16S_AMP
	2	16S_AMP	V4 PCR with 515F/806R constructs containing spacers and sequencing-primer binding sites	V4 PCR1 product
	3	Diluted V4 PCR1 product	Indexing PCR with i5 and i7 primers	Dual-indexed Illumina library
	4	Indexed libraries	Normalization, pooling, 0.5×–1.2× bead size selection, qPCR, and equimolar pooling	Final sequencing pool
	5	Final sequencing pool	MiSeq paired-end sequencing with PhiX spike-in	Raw demultiplexed sequencing data

2

Protocol 1. Full-length 16S rRNA amplification with a *Rickettsia buchneri* blocking primer

2h

3 **Prepare the PCR workspace** 00:10:00

10m

Clean the pre-PCR work surface and assemble reagents on ice.
Label tubes for samples and controls.
Gently mix and briefly centrifuge reagents.

4 **Prepare the full-length 16S PCR reactions** 00:00:00

20m

	A	Stock Concentration	Volume per 50 µL reaction	Final Concentration
	2× Q5 Mastermix	2×	25 µL	1×

	A	Stock Concentration	Volume per 50 µL reaction	Final Concentration
	Tick genomic DNA	~5 ng/µL	3 µL	~15 ng input DNA
	16S-27F-YMY	10 µM	2.5 µL	0.5 µM
	16S-1492R-HY	10 µM	2.5 µL	0.5 µM
	18F-Rb-C3 blocking primer	10 µM	15 µL	3.0 µM
	Nuclease-free water	—	2 µL	—
	Total	—	50 µL	—

- For multiple reactions, prepare a master mix containing all components except template DNA.
- Dispense the required master-mix volume into each labeled tube.
- Add 3 µL of tick genomic DNA to each sample tube.
- Add the corresponding control template or nuclease-free water to control tubes.
- Mix gently and briefly centrifuge.

4.1 **Critical:** The blocking primer is used at six times the volume of amplification primer.

5 Amplify the full-length bacterial 16S rRNA gene with blocking primer

45m

	A	B	C	D
	Initial denaturation	95 °C	2 min	1
	Denaturation	95 °C	30 s	20
	Annealing	62 °C	30 s	
	Extension	72 °C	1 min	
	Final extension	72 °C	10 min	1
	Hold	10 °C	Indefinite	1

SPRI bead cleanup of full-length 16S amplicons

6 **Transfer PCR products**

- Label one 1.5 mL microcentrifuge tube for each sample.
- Transfer the entire 50 µL PCR product to the corresponding tube.

7 **Bind DNA to SPRI beads** 30m

- Add 50 µL SPRI beads to each 50 µL PCR reaction for a 1:1 bead-to-sample ratio.
- Mix thoroughly.
- Incubate at room temperature for 00:10:00 to 00:30:00

8 **Separate beads** 7m

- Briefly centrifuge the tube to collect liquid.
- Place the tube on a magnetic rack for approximately 00:03:00 or until the supernatant is completely clear.
- Carefully remove and discard the supernatant without disturbing the bead pellet.

9 **Wash the beads twice**

- Remove the tube from the magnet and add 200 µL of fresh [M] 80 % (v/v) ethanol.
- Resuspend the beads thoroughly.
- Briefly centrifuge, return the tube to the magnet, and wait until the supernatant is clear.
- Remove and discard the supernatant.
- Repeat the ethanol wash once.

10 **Remove residual ethanol**

- Briefly centrifuge the tube.
- Return it to the magnet.
- Remove the remaining liquid with a small-volume pipette.

11 **Air-dry the beads**

Air-dry until the bead pellet is no longer glossy.

11.1 **Critical:** Do not overdry the beads. Overdrying can reduce DNA recovery

12 **Elute the purified amplicon** *[Timing: 15–30 min]* 45m

- Remove the tube from the magnetic rack.
- Add 16 µL nuclease-free water.
- Resuspend the bead pellet thoroughly.
- Incubate at room temperature for 00:15:00 to 00:30:00 off the magnet.
- Vortex occasionally during incubation.

13 **Recover the eluate**

- Briefly centrifuge the tube.
- Place it on the magnetic rack until the supernatant is clear.
- Transfer 14 µL of clear supernatant to a fresh tube.

14

Expected result

Approximately 14 µL of purified full-length bacterial 16S rRNA amplicon, for downstream V4 library construction.

15 Pause point: You could store the purified 16S amplicons in -4 °C for short term storage

Protocol 2 16S V4 regions amplification

16 **Prepare the PCR workspace** 00:10:00

Clean the pre-PCR work surface and assemble reagents on ice.
Label tubes for samples and controls.
Mix and briefly centrifuge reagents.

17 **Prepare V4 amplicon PCR reactions**

	Reagents	Stock Concentration	Volume per 8 µL reaction
	2× Q5 High Fidelity Mastermix	2×	4 µL
	16S_AMP	—	3 µL
	Forward V4 primer	10 µM	0.5 µL
	Reverse V4 primer	10 µM	0.5 µL
	Total	—	8 µL

- Use PCR strip tubes rather than plates
- Dispense 5 µL of master mix into each corresponding tube.
- Add 3 µL 16S_AMP from the corresponding sample.
- Mix thoroughly and briefly centrifuge.

18 **Amplify the V4 regions**

	A	Temperature	Time	Cycles
	Initial denaturation	95 °C	2 min	1
	Denaturation	95 °C	45 s	15
	Annealing	50 °C	45 s	
	Extension	72 °C	1 min	
	Final extension	72 °C	10 min	1
	Hold	10 °C	Indefinite	1

19 **Dilute the V4 amplicons by 20 folds**

- Add 152 µL nuclease-free water directly to each 8 µL PCR2 product.
- Vortex to mix.
- Briefly centrifuge.

20 Pause point: Diluted PCR2 products can be stored at 4 °C for short-term storage

Protocol 3 Indexing PCR

21 **Prepare the PCR workspace** 00:10:00

Clean the pre-PCR work surface and assemble reagents on ice.
Label tubes for samples and controls.
Mix and briefly centrifuge reagents.

22 **Indexing PCR reaction**

	Component	Stock Concentration	Volume per 10 µL reaction
	Diluted V4 PCR2 product	—	3 µL
	i5 tagging primer, unique to each well	10 µM	1 µL
	i7 tagging primer, one per plate	10 µM	1 µL
	2× Q5 High Fidelity Mastermix	2×	5 µL
	Total	—	10 µL

- Add 3 µL of the diluted V4 PCR2 product
- Add 1 µL of the [M] 10 micromolar (µM) i5 and i7 index primers

- Mix throughly and briefly centrifuge

23 **Indexing PCR conditions**

	A	Temperature	Time	Cycles
	Initial denaturation	95 °C	2 min	1
	Denaturation	95 °C	30 s	10
	Annealing	50 °C	30 s	
	Extension	72 °C	30 s	
	Final extension	72 °C	10 min	1
	Hold	10 °C	Indefinite	1

Protocol 4 : Library Normalization, size selection and quantification

1h 25m

24 **Part A. Plate normalization using Charm Biotech Just-a-Plate 96 PCR Purification and Normalization Kit**

1. Transfer indexed libraries to the normalization plate

- Transfer 10 µL of each indexed library into the corresponding normalization-plate well.
- If evaporation has reduced the available volume below 10 µL, proceed with the remaining volume

25 **2. Add binding buffer**

- Add an equal volume of NB7 binding buffer to each well, typically 10 µL.
- Mix thoroughly.
- Seal the plate, vortex, and briefly centrifuge.

26 **3. Incubate for binding** 1h

Incubate the sealed plate at room temperature for 01:00:00 or longer.

27 **4. Remove binding solution**

Aspirate and discard the liquid without scraping the sides of the wells.

28 **5. Wash the normalization plate twice** *[Timing: 10–15 min]*

- Dispense 50 µL WB2 wash buffer into each well. Confirm that ethanol was added to WB2 before first use.
- Using a multichannel pipette and one set of tips, pipette up and down twice.

- Completely aspirate and discard the wash buffer.
- If necessary, invert and tap the plate on paper towels to remove residual buffer.
- Repeat the wash once.

29 **6. Air-dry the normalization plate** 5m

Air-dry the plate for 00:05:00 .

30 **7. Elute normalized libraries** 20m

- Add 20 µL EB1 elution buffer to each well.
- Seal, vortex, and briefly centrifuge the plate.
- Incubate at room temperature for 00:20:00 .

31 **8. Pool normalized libraries by plate**

- Transfer 15 µL from each well into a reagent reservoir or strip tubes.
- Combine all normalized libraries from one plate into a single labeled microcentrifuge tube.

32 **Pause point:** Normalized DNA may be stored at 4 °C for short-term storage or -20 °C for long-term storage.

33 **Part B. Size selection using Bluepippin for 430bp**

1. Protocol Configuration

- Turn on the bluepippin instruments and its desktop GUI.
- Create a NEW protocol under Protocol Editor
- Select **Cassette Type Selector** and choose the 2% DF Marker V1 option as reference.
- Under **Protocol Parameters**, assign the sample lanes to be run.
- Set the **Mode** dropdown for your sample lanes to **Range** between 380 bp to 500 bp.
- Save the protocol.

34 **2. Cassette Preparation**

- Remove the pre-cast [M] 2 Mass / % volume agarose gel cassette from its foil pouch and inspect all the reservoirs and lanes for buffers. If the spare buffers were evaporated (i.e. empty of half filled), fill the reservoirs with spare buffer.
- Make sure there is no air bubbles trapped in the elution side.
- Place the cassette in the instrument's optical nest.
- Peel off the white adhesive tapes and pippette out the buffer from the elution well and replace it with 80 µL of fresh buffer
- Close the lid and start the continuity test to ensure all lanes are passed.

35 **3. Sample Preparation and Loading**

- Aliquot the samples to 30 µL into a fresh 1.5 mL eppendorf tubes.
- Add 10 µL of loading marker.

- Mix thoroughly by pipetting.
- Pipette in the 40 µL of prepared sample into the input well on the cassette.
- Close the lid and, load the protocol and start the run.

36 **4. Sample recovery**

After the run ends, carefully pipet out the size-selected fragments from the elution buffer and transfer into clean 1.5 mL eppendorf tube.

37 **Part C qPCR library quantification using NEB library quantification kit**

1. Prepare 1:1,000 and 1:10,000 library dilutions

- Add 999 µL ddH2O to a fresh 1.5 mL tube.
- Add 1 µL purified library and mix thoroughly to obtain a 1:1,000 dilution.
- Add 90 µL ddH2O to a second tube.
- Transfer 10 µL of the 1:1,000 dilution into the second tube and mix to obtain a 1:10,000 dilution.

38 **2. Prepare the NEB library quantification reagents**

- Thaw the NEB E7630 kit components on ice.
- Vortex and briefly centrifuge all tubes.
- If the primer mix has not yet been added to the master mix, add it and label the master mix accordingly.
- Follow the NEB and qPCR-instrument manuals when using an instrument other than the Bio-Rad CFX96.

39 **3. Set up qPCR reactions in triplicate**

Prepare triplicate reactions for each of the six standards and for both dilutions of each purified library pool.

	Component	Volume per 15 µL qPCR reaction
	Master mix plus primer pool	12 µL
	Standard or diluted library	3 µL
	Total	15 µL

40 **4. Run library-quantification qPCR**

	A	Temperature	Time	Cycles
	Initial denaturation	95 °C	1 min	1
	Denaturation	95 °C	15 s	35

	A	Temperature	Time	Cycles
	Annealing/extension	63 °C	45 s	

41 **5. Confirm library size**
After qPCR, select representative libraries and run them on an agarose gel to confirm library size.

42

Expected result

Expected result: Approximately size of V4 library is 430 bp and 400 bp as the NEB standard size.
